# Territorial and Non‐Territorial Subpopulations of Wilson's Warblers Return to Central California Breeding Grounds in Two Migratory Waves

**DOI:** 10.1002/ece3.70672

**Published:** 2024-12-04

**Authors:** William M. Gilbert

**Affiliations:** ^1^ Department of Science and Mathematics Chabot College Hayward California USA

**Keywords:** adaptive breeding strategy, *Cardinella pusilla*, cloacal protuberance, cost‐minimizing dates, floater, migratory return, non‐territorial, reproductive success, territoriality, Wilson's Warbler, Reinita de Wilson, *Cardinella pusilla*, flotador, no territorialidad, territorialidad, retorno migratorio, éxito reproductivo, protuberancia cloacal, estrategia de cría adaptativa, fechas minimizadas de costos

## Abstract

Early migratory return for territorial passerines is important, since earlier return results in better territories, and greater reproductive success. Many passerine studies have found that migratory return dates for first‐breeding‐season (SY) individuals was a week or less later than for older (ASY) birds. Many of both ASY and SY birds in these studies became territorial, although more SY than ASY birds became non‐territorial floaters. In this study of Wilson's Warblers, however, nearly all individuals returning early and becoming territorial were ASY birds, and very few were SY birds. However, second waves of returning Wilson's Warblers, containing both SY and ASY birds, returned about 3–5 weeks after the first migratory waves, and no males in those second waves ever became territorial. No later‐returning, second‐wave female initially ever became territorial either, although some subsequently became replacement mates for earlier‐returning males. Some male Wilson's Warblers were territorial, and others were non‐territorial, during all of their study‐area years. However, some males switched status over successive years, and migratory return when non‐territorial always was several weeks later than when territorial. Findings from this study indicated, for some males during some years, that non‐territoriality was more adaptive than was territoriality. This study's finding, of 3–5 week later migratory return waves for non‐territorial, than for territorial, Wilson's Warblers, supports findings by Stewart (1973), that Wilson's Warblers return to breeding grounds in two migratory waves. Findings from both studies may provide empirical evidence supporting Kokko's (1999) hypothesis that migratory birds, not competing for territories, should return to breeding grounds later, on their “cost‐minimizing dates.”

## Graphical abstract

In this study of Wilson's Warblers, nearly all individuals returning early and becoming territorial were ASY birds, and very few were SY birds. However, second waves of returning Wilson's Warblers, containing both SY and ASY birds, returned about 3–5 weeks after the first migratory waves, and no males in these second waves ever became territorial.

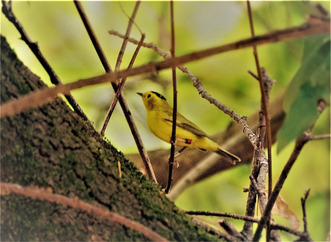

## RESUMEN

El retorno migratorio temprano de los paseriformes territoriales es importante, ya que el retorno temprano resulta en mejores territorios y mayor éxito reproductivo. Muchos estudios de paseriformes han encontrado que las fechas de regreso migratorio para los individuos de la primera temporada de reproducción (SY) fueron una semana o menos tarde que para las aves más viejas (ASY). Muchas de las aves ASY y SY en estos estudios se volvieron territoriales, aunque más aves SY que ASY se convirtieron en flotadores no territoriales. Sin embargo, en este estudio de las currucas de Wilson, casi todos los individuos que regresaban temprano y se volvían territoriales eran aves ASY, y muy pocos eran aves SY. Sin embargo, las segundas oleadas de currucas de Wilson que regresan, que contenían aves SY y ASY, regresaron entre tres y cinco semanas después de las primeras olas migratorias, y ningún macho en estas segundas oleadas llegó a ser territorial. Ninguna hembra de la segunda ola que regresó más tarde inicialmente se volvió territorial, aunque algunas posteriormente se convirtieron en parejas de reemplazo para los machos que regresaron antes. Algunos machos de la curruca de Wilson fueron territoriales, y otros no territoriales, durante todos sus años de área de estudio. Sin embargo, algunos machos cambiaron de estatus a lo largo de los años sucesivos, y el retorno migratorio cuando no eran territoriales, siempre era varias semanas más tarde que cuando eran territoriales. Los resultados de este estudio indicaron que, para algunos machos durante algunos años, la no territorialidad fue más adaptativa que la territorialidad. El hallazgo de este estudio, de tres a cinco semanas más tarde en las olas de retorno migratorias para las currucas de Wilson no territoriales, que para las territoriales, apoya los hallazgos de Stewart (1973), de que las currucas de Wilson regresan a las zonas de reproducción en dos oleadas migratorias. Los resultados de ambos estudios pueden proporcionar evidencia empírica que apoye la hipótesis de Kokko (1999), de que las aves migratorias que no compiten por territorios deberían regresar a las zonas de reproducción más tarde en sus “fechas de minimización de costos.”

## 1 Introduction

In agreement with the generally recognized importance of territoriality for reproductive success in migratory, territorial bird species, prior studies have found strong selective pressure for individuals of such species to return to breeding grounds as soon as possible to establish, or be included on, high‐quality breeding territories (Kokko 1999; Cooper et al. 2009; Lerche‐Jørgensen et al. 2018). However, populations of migratory, territorial species often additionally contain subpopulations of non‐territorial “floaters” (Hensley and Cope 1951; Stewart and Aldrich 1951; Newton 1992, 1998), which generally exist outside of breeding territories. These floaters have been considered weaker, less fit, less experienced, and/or otherwise less capable individuals which are less able to compete for breeding territories. They also may be less capable of returning to breeding grounds early, and less able to survive once they do return (Brown 1969; Winker 1998; Penteriani, Ferrer, and Delgado 2011; Newton 1992, 1998). For these reasons, migratory return times of floaters in such species would be expected to be later than those of territorials.

However, the migratory return dates of territorials versus floaters have not been empirically investigated for any passerine species, to the best of my knowledge. The principal reason for this is the difficulty of determining migratory return dates for floaters. While such dates for territorials are easily determined, based on singing and/or sighting of newly returned males and females on breeding territories, returning male floaters generally do not sing, and both male and female floaters have been characterized as “cryptic,” and occupying a non‐territorial “underworld” within territorial breeding populations (Smith 1978). Even so, since floater passerines tend to be younger, first breeding season (second year, or SY) individuals, while territorial passerines tend to be older, second or later breeding season (after‐second‐year, or ASY) individuals (Hill 1988; Lozano, Perreault, and Lemon 1996; Hahn and Silverman 2006; Cooper et al. 2009; Moreno 2016), an approximation of migratory return times for passerine floaters versus territorials may be seen in studies comparing migratory return, or migratory passage times, based on age (Hill 1988; Francis and Cooke 1986, 1990; Stewart, Francis, and Massey 2002; Hahn and Silverman 2006; Cooper et al. 2009; Lenda, Maciusik, and Skórka 2012; Moreno 2016). Such studies have found, for many species, that SY individuals tend to return to breeding grounds, or pass through banding stations, from a few days to a little over a week later than ASY individuals, and at most about 2 weeks later (Hill 1988; Lozano, Perreault, and Lemon 1996).

The differential migratory return times to breeding grounds of territorial versus floater, or of ASY versus SY, migratory passerines are of interest relative to a game‐theoretic model developed by Kokko (1999). That model essentially states that, if a bird has no reason to return to breeding grounds as a function of establishing a breeding territory, but only as a function of better foraging and survival, its migratory return time should correspond closely to the individual's “cost‐minimizing date.” That date would be when environmental constraints on breeding grounds, such as poor food supply or poor weather, were minimal.

It can be presumed that the negative environmental consequences of early migratory return, suggested in Kokko's (1999) model, would have affected SY birds that prior‐cited studies found returned early to breeding grounds. Driven by a selective pressure to attain reasonable reproductive success, however, which required territoriality, the SY birds returned to breeding grounds earlier than their hypothesized cost‐minimizing dates, in spite of less‐than‐optimal environmental conditions, less ability than ASY birds to deal with those conditions, and less ability than ASY birds to compete for territories. Even given these environmental and competitive difficulties, however, early migratory return presumably does benefit many SY birds, as many do achieve territoriality and do raise broods (Hill 1988; Lozano, Perreault, and Lemon 1996; Hahn and Silverman 2006; Cooper et al. 2009; Moreno 2016). However, the percentage of non‐territorial floaters among SY subpopulations is higher, the quality of breeding territories obtained is lower, and/or the reproductive success of the SY birds is lower, than for ASY birds (Holmes, Marra, and Sherry 1996; Lozano, Perreault, and Lemon 1996; Bayne and Hobson 2002).

**FIGURE 1:**
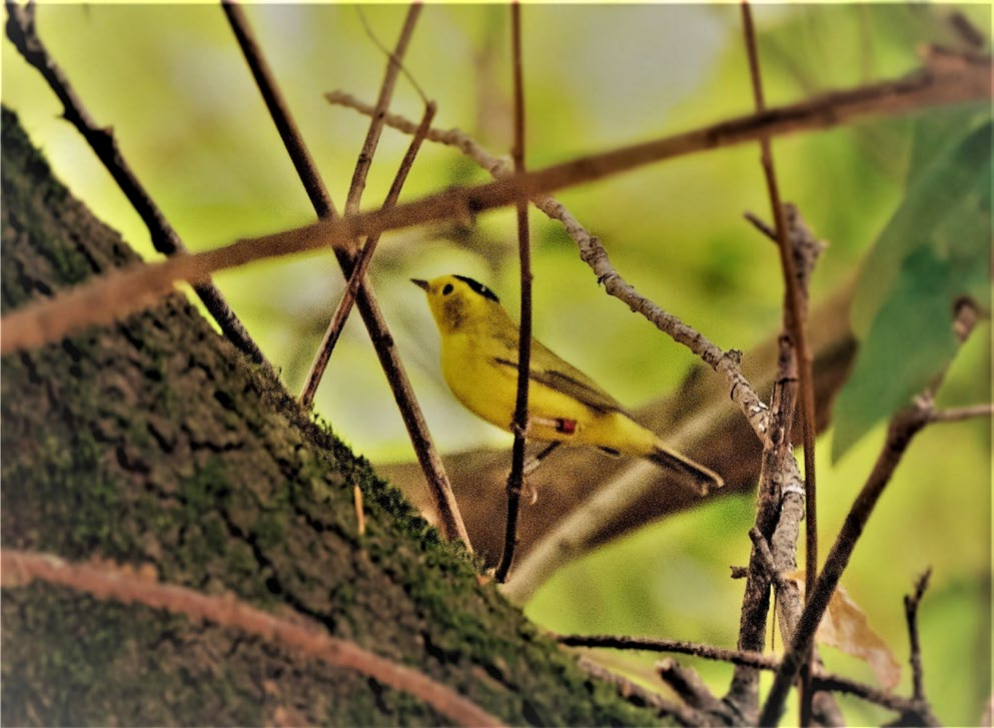
A color‐banded male Wilson's Warbler in the study area at the Tilden Nature Area in the East San Francisco Bay Area of central inner‐coastal California, U.S.A. Photograph taken in the wild by Erica Kawata.

I here report, based on a 13‐year study of Wilson's Warblers (Figure 1), that mean migratory return dates to breeding grounds for non‐territorial males and females in my study population were significantly about 3 to over 5 weeks later, varying with the method of determination, than were mean return dates for respective sexes of territorials. These differences in mean migratory return dates were up to several weeks greater than comparative differences between ASY and SY subpopulations reported for other passerine species (Francis and Cooke 1986, 1990; Lozano, Perreault, and Lemon 1996; Stewart, Francis, and Massey 2002; Hahn and Silverman 2006; Cooper et al. 2009).

Findings from this study also show that non‐territorial subpopulations of Wilson's Warblers returned to breeding grounds, not just significantly later than territorials, but also in distinctly later migratory waves. These migratory waves were separated by time gaps during which few birds returned. A previous study, that of Stewart (1973), similarly reported Wilson's Warblers returning to breeding grounds in two migratory waves. The later mean return dates that both Stewart (1973) and I found for second migratory waves of Wilson's Warblers may correspond to cost‐minimizing dates for the species, as theorized by Kokko (1999). I found that no second‐migratory‐wave male Wilson's Warbler, whether of SY or ASY age, ever formed a Type A breeding territory (Nice 1941, that is, a territory in which all breeding and foraging activities occur), even when habitat suitable for such activities appeared to be available.

Additional findings of this study are that, in contrast to ASY males, just two SY male Wilson's Warblers returned early from migration and became territorial, that some males switched from territorial to non‐territorial status, or vice versa, among breeding seasons, and that the migratory return dates for those males, when non‐territorial, always were several weeks later than the dates when they were territorial. In this study, I refer to later‐returning Wilson's Warblers that did not establish Type A breeding territories as “non‐territorials” rather than “floaters.” The reason for this distinction is that floaters characteristically have been considered to be birds that are “seeking” to become territorial, and indeed studies have shown that they do become territorial when territories become available (Hensley and Cope 1951; Stewart and Aldrich 1951; Newton 1992, 1998). No later‐returning, non‐territorial male Wilson's Warbler in this study ever became territorial, however, even when apparently suitable territorial habitat remained available, nor even when, on rare occasions, established territories and previously mated females became available.

## 2 Methods

### 2.1 Study Area, Timing, and Effort of Observation, Monitoring Nest Activity, and Color‐Banding

From 1997 through 2010, exclusive of year 2000, I conducted a comprehensive field study of Wilson's Warblers at a study site in the Tilden Nature Area, in the hills of the East San Francisco Bay, to the east of the City of Berkeley, California, U.S.A. My study site, of approximately 4.7 ha, included both riparian habitat along the Wildcat Creek valley, and oak/bay woodland habitat extending uphill to the west. More information regarding the study site can be found in Gilbert (2022). Among other information, I recorded migratory return dates of territorial male and female Wilson's Warblers, as well as the return dates and number of sightings of individuals that I considered to be non‐territorial males and females.

I initiated observations for migratory returning male Wilson's Warblers each year in early March, prior to the time that first male Wilson's Warblers returned from migration, and carried out observations late into the summer, generally into August. However, for purposes of this study, only observations through 9 May were relevant, as that time length incorporated all migratory return information for both territorials and non‐territorials needed for this study. I surveyed the entire study area each day of each year (except when raining) during the relevant migratory return period prior to 10 May. These daily surveys allowed a complete assessment of when each territorial male arrived each year (usually based on singing), and when territorial females arrived (usually based on when males temporarily stopped singing). Daily surveys later in the breeding season verified continued territory occupancy by territorial males. More importantly, however, these surveys detected nest‐building events by territorial and non‐territorial females. Daily surveys also detected “exploring” events by non‐territorial females, where those females would move through undergrowth over extended time, and often would pick up, but then drop, vegetation. I refer to both nest building and exploring behaviors as “nest activity,” and such nest activity often induced intrusion by males into areas where it was happening.

During the study, I was able to locate and observe only a fraction of the nest activity events that no doubt occurred. However, those relatively few events allowed monitoring opportunities that provided information on intrusion by territorial males (Gilbert 2024). Also, of importance to this study, the timing of territorial male occupancy, and the timing of non‐territorial male intrusion, at nest activity sites were the main sources of information for migratory return dates.

I annually netted and color‐banded (BBL permit #22521) many territorial males, and a lesser proportion of territorial females, occupying breeding territories. I also color‐banded lesser numbers of individuals that I determined to be non‐territorial males and females. The total number of Wilson's Warblers that I color‐banded over 13 years, from 1997 through 2010, exclusive of year 2000, was 117 males and 49 females. Of those color‐banded totals, 93 males and 37 females were territorials, while 24 males and 12 females were non‐territorials. Banding of subjects was based on opportunity and occurred in relation to the importance of other field activities. I attempted to color‐band most territorial males, with assistance from recorded playback, and succeeded with a majority of territorial males. I banded non‐territorial males and females less frequently, and more often with set nets around areas of nest activity, and in Current Grove Woods (see below).

### 2.2 Determining Status and Migratory Return Dates for Territorials and Non‐Territorials

#### 2.2.1 Determinations for Territorials and Color‐Banded Non‐Territorials

I easily determined the territorial status of territorial males and females, based on their occupancy of specific breeding territories. I based the location of those breeding territories on areas and limits in which I sighted and heard territorial males singing. I did not map limits of territories, as that was not a goal of this study. However, prior observation (Ammon and Gilbert 2020) of territorial behaviors had shown that male Wilson's Warblers are protective of their spaces, and aggressively drive intruders, including neighboring males, from their territories. Resident male (RM) territories were well separated in this study. Daily surveys confirmed respective males, whose song patterns tended to be distinctive, consistently singing from their respective territories, and neighboring males not singing from those territories. I determined the migratory return dates of territorial males based on when I first sighted the birds on their territories in the spring or, more often, when I first heard them sing. Female migratory returns coincided with the initial termination of a male singing on its territory, since a male ceased advertising for a mate when a female entered its territory, and did not resume singing for a period of time if the female remained.

I color‐banded a number of netted males and females that I initially considered were likely non‐territorials, and I subsequently confirmed their non‐territorial status by not sighting them as banded occupants of any territory, while sometimes sighting them in non‐territorial areas. I determined approximate migratory return dates for those color‐banded non‐territorials based on when I first sighted them in a given year.

#### 2.2.2 Determining Non‐territorial Status, and Migratory Return Dates, for Unbanded Non‐territorial Males, Based on Behaviors

Additional to color‐banded non‐territorials, many unbanded males and females, sighted mostly outside of breeding territories, also were likely non‐territorials. Confirming their status and approximate migratory return dates largely was based on behaviors. Starting about a month after the beginning of the breeding season, I frequently observed closely spaced (< 0.3 m) groups of males intruding into sites of female nest activity. I called these closely spaced intruding groups of males “GANGs” (“Gregarious all‐male non‐territorial groups”). These GANGs usually numbered three or four males, but sometimes more, and most members I saw in these groups were unbanded. Those seen to be banded, with one exception, were known color‐banded non‐territorial males. Since the majority of territorial males were color‐banded, but I saw only one participating in an intruding GANG, I concluded that most males in these intruding groups likely were not‐territorial males. Sighting the earliest of those closely spaced intruding male GANGs, in addition to initially sighting color‐banded non‐territorial males, provided estimates of the earliest non‐territorial male migratory return dates.

**FIGURE 2:**
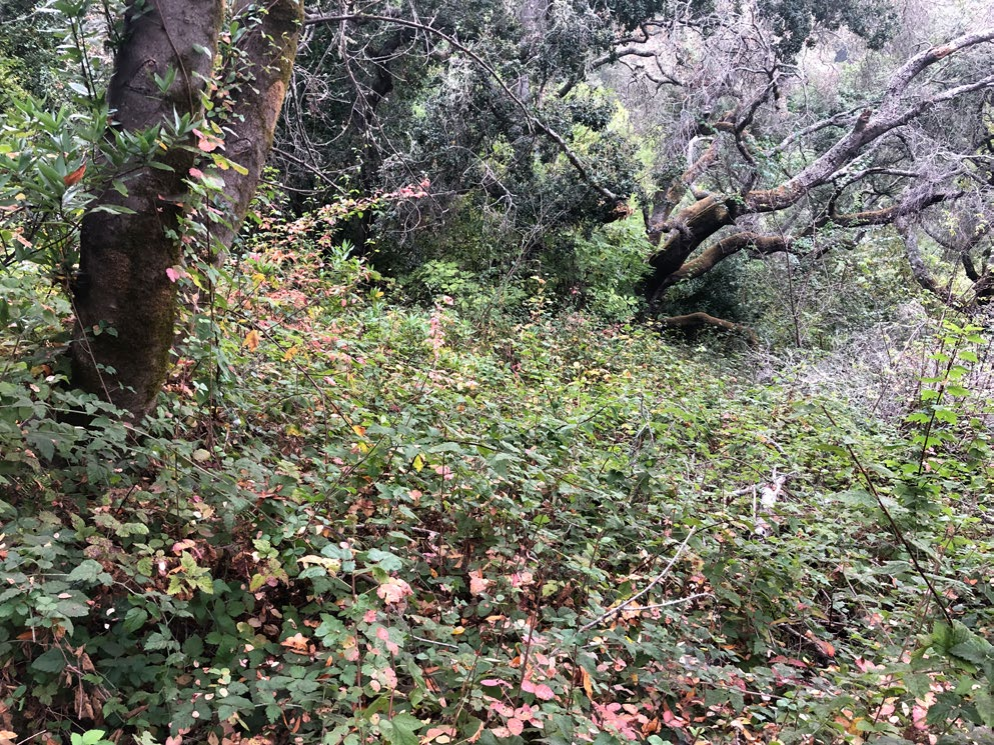
A transient group interaction, observed on May 13, 1997, and drawn from memory. When observed, this interaction proceeded relatively slowly through low vegetation. However, if the female being pursued by multiple males flew away, males in the pursuing group coalesced into a compact group and rapidly followed her. Spatial relationships of the illustration are approximately accurate, but sizes of individual birds, as illustrated, are larger than reality.

Additional to sighting GANGs of non‐territorial males intruding into areas of female nest activity, I also occasionally sighted closely spaced GANGs pursuing isolated females over great distances. I called these mobile events “transient group interactions” (Figure 2; Ammon and Gilbert 2020). As was similar for most males sighted in territory‐intruding GANGs, the pursuing males in these transient group interactions all were seen to be unbanded. It follows that most, if not all, likely were non‐territorials, since a majority of territorial males were color‐banded. Sightings of transient group interactions were too infrequent, however, to be used for estimating migratory return dates of non‐territorials.

Contrasting with male group behaviors of non‐territorial GANGs, territorial males did not form closely spaced groups pursuing fertile females. Territorial males, with just one sighted exception, only intruded singly into areas of female nest activity, and never formed closely spaced groups composed entirely of territorial males. Most often I sighted male owners of territories, if they were adjacent to areas of female nest activity, position themselves at the borders of their own territories, and as close as possible to the females displaying nest activity, and sing persistently.

#### 2.2.3 Determining Non‐Territorial Status, and Migratory Return Dates, for Unbanded Non‐Territorial Females, Based on Behaviors

**FIGURE 3:**
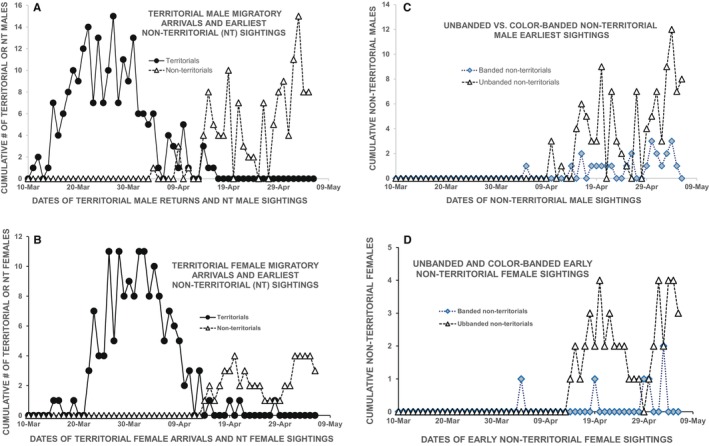
A small portion of “Current Grove Woods,” showing the predominant understory of low shrubs in the foreground, bordered by mature oak‐bay woodland seen in the background. This area only once hosted a Type A breeding territory, but hosted a disproportionate number of non‐territorial Wilson's Warbler females displaying nest activity. It also was an area where non‐territorial males often intruded into areas of female nest activity, and sometimes attached to non‐territorial females to guard their nest building and/or help feed their nestlings.

As with non‐territorial males, observation of unique behaviors of non‐territorial females could be used to separate them from territorial females. These non‐territorial female behaviors mostly involved nest activity, such as extended exploring in undergrowth, and picking up and dropping nest material. By way of contrast, I never observed territorial females involved in such prolonged nest activity. Territorial females proceeded with nest building with no observed trial behaviors, although they characteristically did require longer time intervals for building their initial nests than for building replacement nests following nest predation. Additional to their unique nest activity behaviors, I identified females as being non‐territorial based on their frequently occupying a portion of the study area of approximately 0.5 ha that I called “Current Grove Woods” (Figure 3). That area was mainly filled with low‐growing shrubs and undergrowth, and was a “woods” only relative to a more uphill area that I called the “Current Grove.” Current Grove Woods only once hosted a Wilson's Warbler breeding territory, and the area apparently was subprime habitat for Type A Wilson's Warbler breeding territories. However, I annually sighted relatively large numbers of non‐territorial males and females in Current Grove Woods, and saw a substantial proportion of non‐territorial nest activity there. Sighting the earliest annual female nest activity in Current Grove Woods, as well as in other non‐territorial areas of the study site, provided estimates of annual non‐territorial female migratory return.

### 2.3 Determining ASY and SY Ages in Wilson's Warblers, and the Relationship of Those Ages to Wilson's Warbler Territorial Status

I aged some, but not all, territorial and non‐territorial Wilson's Warblers based on two processes. Initially, I aged some netted birds as SY or ASY when I color‐banded them, based on feather data, that is, the relative wear of remiges and/or molt limits (Pyle 1997). Secondly, I logically concluded that some color‐banded individuals were ASY birds if I sighted them during their second or later years, regardless of whether I had aged them at initial banding. I compared the numbers of aged males and females with their territorial or non‐territorial status, and present the information in a contingency table.

### 2.4 The Relationships of Wilson's Warbler Age and Status to Those of Other Passerine Species

No previous study of a short‐lived avian species, to the best of my knowledge, has been able to evaluate mean migratory return times for territorial versus non‐territorial birds. The main reason for this has been the difficulty in determining the non‐territorial status of floaters (Cooper et al. 2009), since floaters tend to be cryptic and difficult to observe (Smith 1978). However, several studies have determined the mean migratory return, or migratory passage dates, for older, after‐second‐year (ASY) birds versus younger, second‐year (SY) birds (Hill 1988; Francis and Cooke 1986, 1990; Stewart, Francis, and Massey 2002; Hahn and Silverman 2006; Cooper et al. 2009). These studies have documented that ASY birds characteristically return earlier from spring migration than do SY birds, and the ASY birds typically become territory holders, while a greater percentage of SY birds become floaters. Thus, while I could not compare migratory return dates of Wilson's Warbler territorials and non‐territorials with return dates for territorials and floaters in other species, I could use the return dates of ASY and SY subpopulations, determined in some other studies, as surrogates for the return dates of their territorial and floater subpopulations.

### 2.5 Flexibility in Territorial and Non‐Territorial Status Among Males, and Among Years

Different male Wilson's Warblers in my study had different histories of territorial and/or non‐territorial status over time. This flexibility in status also could be associated with variability in SY or ASY ages, and was related to variable migratory return times. I determined five categories of flexibility based on territorial status and age among male Wilson's Warblers, and based on sighting individuals for at least 2 years. In the few cases where I did not sight an individual male during 1 year, I assumed its status to have been the same as it was in the prior and subsequent year when I did sight the bird. The five categories of territorial and age flexibility, with number of observed examples, were (1) in excess of 20 males that were ASY, or age unknown, and territorial when first encountered, and that remained territorial for the remainder of their study area tenures, (2) one male that was SY and territorial when first encountered, and that remained territorial for the remainder of its study area tenure, (3) 10 males, SY, or age unknown, that were non‐territorial when first encountered, and that remained non‐territorial for the remainder of their study area tenures, (4) five males, SY, ASY, or age unknown, that were non‐territorial when first encountered, but later became territorial, and remained territorial, for the remainder of their study area tenures, and (5) two males, age unknown, that were territorial when first encountered, but became non‐territorial after being displaced from their breeding territories by other males, and remained non‐territorial for the remainder of their study area tenures.

### 2.6 Further Information on Determining Mean Migratory Return Dates for Territorial and Non‐Territorial Wilson's Warblers

#### 2.6.1 Determination for Territorials

As stated above, I determined migratory return dates for territorial male and female Wilson's Warblers based on when they first returned to breeding territories. I determined means and ranges for respective territorial male and female migratory return dates, based on cumulative sightings over 13 years, from 1997 through 2010, exclusive of year 2000. The time extent over which I respectively recorded migratory returns for territorial males and females over the 13 study seasons was about a month and a half for each sex (see Section 3). I call these total migratory return intervals the inclusive migratory return intervals, and I used these inclusive intervals for plotting graphs of migratory return. However, I considered the mean migratory return dates for territorial males and females to be more accurately determined by just considering time periods over which most individuals returned, since inclusive time intervals contained small numbers of early and late single outlier values. I thus calculated mean migratory return dates based on the more restricted time intervals, which I called the effective time intervals. For territorial males, the effective time interval was a migratory return period over which a cumulative 94.8% (199/210) of males returned from migration, and for territorial females, it was a migratory return period over which a cumulative 95.6% (154/161) of territorial females returned from migration.

#### 2.6.2 Determinations for Non‐Territorials

I recorded, on each calendar day, the number of sightings of males and females that I considered were non‐territorial, based on sighting non‐territorials that were color‐banded, or sighting individuals with behaviors characteristic of non‐territorials. I assumed that migratory return dates for non‐territorials extended over the same effective time intervals that I had determined for territorial males and females, respectively. I assumed that the appropriate return intervals for non‐territorial males and females, and thus, the return intervals from which I determined their mean migratory return dates, started from the first dates that I respectively sighted non‐territorial males and females in my study area. I made determinations of mean migratory return dates for non‐territorial males and females based on samples that included some color‐banded birds, but a greater proportion of unbanded birds.

#### 2.6.3 Comparative Accuracy of Mean Migratory Return Dates Determined for Territorials and Non‐Territorials

While determining mean migratory return dates for territorial males and females was easy based on sighting or hearing them in their respective territories, obtaining estimates of migratory return dates for non‐territorial males and females was more problematic. Migratory return for both sexes of non‐territorials had to be based on sightings. It follows that “mean migratory return dates” for non‐territorials actually reflected their “mean early‐sighting dates.” For purposes of this study, however, these mean early‐sighting dates for non‐territorials likely were sufficiently close to their actual mean migratory return dates. The difference between determined mean migratory return dates for territorials and non‐territorials was so robust that possible errors based on not having determined more exact migratory return dates for non‐territorials likely was irrelevant to the study's essential purposes. Importantly, the essential purposes of this study was not to determine exact mean migratory return dates for either territorials or non‐territorials, but to determine if the mean migratory return dates of those two subpopulations were statistically different, and if so were sufficiently separated to constitute distinct waves of migratory return.

### 2.7 Additional Ways I Analyzed Migratory Return Dates for Territorial and Non‐Territorial Wilson's Warblers

#### 2.7.1 Determination of Means Based on Earliest‐Returning Males

Additional to determining the mean migratory return dates for large samples of territorial and non‐territorial birds, I calculated and compared the mean return date for the five earliest‐returning territorial males with the mean return date for the five earliest‐returning non‐territorial males. A similarity in results based on this second methodology, and that based on the total samples of males, would validate results for both methods, while a dissimilarity would indicate that results from one, or possibly both procedures, were in error. The paucity of reliable earliest return dates for non‐territorial females disallowed a similar comparison for females.

#### 2.7.2 Determination of Means Based on Males With Flexible Territorial Status Among Years

A second additional method for determining mean migratory return dates was based on males that had both territorial and non‐territorial status during different years. I compared the mean migratory return dates during years when such males were territorial versus the years when they were non‐territorial. Females did not show territorial flexibility among years, or I did not detect it, and so were not analyzed based on this method.

#### 2.7.3 Determinations of First Male Migratory Return Dates on a Year to Year Basis

I tabulated the first migratory return dates for territorial and non‐territorial males during each of the 13 years of the Study. This analysis tested whether differences in mean territorial and non‐territorial migratory return dates might have resulted from aberrant conditions found during a restricted number of years, or whether differences in means resulted from differences in return dates of the two subpopulations that were consistent year to year.

#### 2.7.4 Testing the Proportional Relationships of Male and Female Territorial and Non‐territorial Samples Versus Their SY or ASY Ages

I carried out contingency analyses to test if male and female Wilsons Warblers had different proportions of territorial versus non‐territorial subpopulations based on SY or ASY ages. These analyses also provided information on the frequencies of the two age classes found in the respective status groups.

### 2.8 Statistical Methods

I statistically analyzed the tabular information for territorial and non‐territorial subpopulations, using the MS Excel statistics program in MS Office 2019, MS 360 cloud, and/or the Instat statistical package. I compared total territorial and non‐territorial data samples with Student's t tests. I analyzed small data samples, such as for the five earliest‐returning males, with the Mann–Whitney U test. I compared total versus color‐banded non‐territorial male samples to see if they showed similar proportions for early versus later migratory return, using the Chi‐square test. I used the 0.05 probability level for statistical significance in all comparisons, and state probability results for data analyses in respective tables.

### 2.9 Plotting Migratory Return Dates for Territorials and Early Detection Dates for Non‐Territorials

I plotted, on separate scatterplot graphs for males and females, the migratory return dates of territorials, and the early‐detection dates of non‐territorials. I plotted the return dates for territorials over the entire inclusive return times for both males and females. For non‐territorials, I plotted early detections dates, which approximated migratory return dates, over shorter time periods, since inclusive spans of their early‐detection dates could not be determined. However, the plots for non‐territorials covered the same more‐restricted effective time lengths over which I recorded most territorial migratory returns.

### 2.10 Plotting Unbanded Versus Color‐Banded Non‐Territorials

I also plotted, on the same graphs, the early‐detection dates for samples of unbanded versus color‐banded non‐territorials. These combined graphs, plotted for male and female non‐territorials separately, were visual checks for the validity of the migratory return dates for the unbanded samples, whose status had been determined by behaviors, compared with the greater certainty of status based on color‐banded birds. I plotted all graphs, showing relationships of migratory return for territorial and non‐territorial subpopulations, using the MS Excel graphing program in MS Office 2019, and MS 360 cloud.

### 2.11 “Natural” Mate‐Removal Experiments

The classical means by which past researchers have evaluated the presence of non‐territorial floaters in breeding populations has been through mate‐removal experiments (Hensley and Cope 1951; Stewart and Aldrich 1951; Hogstad 1989; Newton 1992, 1998). In this study, time limitations did not allow mate‐removal experiments to see if Wilson's warbler territories might be reoccupied by non‐territorial males. However, two “natural” mate removal experiments occurred during the study. In the first case, a male apparently disappeared completely from its territory for an unknown reason, after the loss of a pair's initial brood. In the second case, a male ceased brood care activity and temporarily disappeared from sight in its territory as an apparent result of a leg injury.

## 3 Results

### 3.1 Tabular Analysis of Migratory Return Dates for Territorials and Non‐territorials

Tables 1a,b state the range and means for migratory return dates during cumulative 27‐day and 23‐day effective migratory return intervals for territorial and non‐territorial male and female samples respectively. The mean migratory return date for territorial males, and the mean date for territorial females, were significantly earlier, by 31 and 28 days, respectively, than the mean first detection dates for non‐territorial males and females. Probability values for these and other tabular comparisons are stated in the tables.

**TABLE 1:** (a, b) Ranges, means, and sample sizes for migratory return dates of territorial and non‐territorial male and female Wilson's Warblers over the effective migratory return periods for each territorial status group. Migratory return dates for non‐territorial males and females were approximated based on early encounter dates of non‐territorials in the study area, usually in non‐territorial areas, or intruding as male GANGS into areas of female nest building or exploring. Probabilities that mean migratory return dates are the same for territorial and non‐territorial samples of either sex were < 0.0001, based on Student's *t*‐tests. (c, d) Ranges and means of migratory return dates for male territorial and non‐territorial Wilson's Warblers, based on two different analytical methods. The first method (c) compares the migratory return dates of the five earliest‐returning males in each territorial status group, and the second method (d) compares the migratory return dates for males that were both territorial and non‐territorial during their tenures at the study site. As in (a,b), migratory return dates for non‐territorial males are approximated from early encounter dates. Probability that the mean migratory return dates for the two status groups were the same was < 0.0001, based on Mann–Whitney *U* analyses.

	(a) Males		(b) Females	
	Territorial	Non‐territorial	Territorial	Non‐territorial
Effective range	15 March–10 April (27 days)	10 April–6 May (27 days)	22 March–13 April (23 days)	17 April–9 May (23 days)
Effective mean ± SD	26 March ± 6.48 days	26 April ± 7.45 days	1 April ± 5.41 days	29 April ± 7.24 days
Difference in mean dates	31 days earlier	31 days later	28 days earlier	28 days later
Sample sizes	199	154	149	62

	(c) Earliest five dates		(d) Same male with alternate status	
	Territorial	Non‐territorial	Territorial	Non‐territorial
Range	11 March–15 March	5 April–12 April	21 March–23 April	5 April–12 June
Mean ± SD	13 March ± 1.6 days	9 April ± 2.6 days	31 March ± 9.2 days	10 May ±22.7 days
Sample size	5	5	12	12

Table 1c shows the mean migratory return date for the five earliest‐returning territorial males compared with the mean migratory return date for the five earliest‐encountered non‐territorial males. The mean early return date for the territorial sample was significantly earlier, by about 27 days, than the mean early‐detection date for the non‐territorial sample. The earliest territorial male arrived 15 days earlier than the earliest‐detected non‐territorial male.

Table 1d shows the respective mean migratory return dates for males that were variably territorial and non‐territorial over their tenures in the study area, as indicated in status categories 4 and 5 described in Section 2. The mean migratory return date when territorial was significantly earlier, by about 31 days, than the mean first‐detection date when non‐territorial. The earliest migratory return date when a male was territorial was 15 days earlier than the earliest first‐detection date when the male was non‐territorial.

Table 2 shows first migratory return dates for territorial and non‐territorial male Wilson's Warblers each year over the 13 years of the study. First migratory return dates for non‐territorials were approximated based on first encounter dates. Results show that first encounter dates for non‐territorial males each year ranged from 5 to 40 days later than for territorial males, but during many years, the earliest non‐territorial males were detected about a month later than the respective migratory return dates of territorial males. Table 2 also indicates that environmental conditions during any given year may have simultaneously affected migratory return dates for both territorial and non‐territorial subpopulations. Thus, the latest migratory return date for territorial males (31 March) and the latest return date for non‐territorial males (5 May) both occurred in 2006. Interestingly, the latest 31 March return date for territorial males (in 2006) still was earlier than the earliest 5 April return date recorded for non‐territorial males (in 1999 and 2002).

**TABLE 2:** Annual dates of first territorial male Wilson's Warbler migratory returns and first non‐territorial male encounters over the 13‐year study.

Year	1997	1998	1999	2001	2002	2003	2004	2005	2006	2007	2008	2009	2010
Territorial	17 March	15 March	26 March	19 March	18 March	22 March	16 March	11 March	31 March	17 March	18 March	18 March	16 March
Non‐territorial	16 April	15 April	5 April	12 April	5 April	22 April	19 April	20 April	5 May	18 April	22 April	15 April	22 April

### 3.2 Plotting of Territorial and Non‐Territorial Migratory Return

**FIGURE 4:**
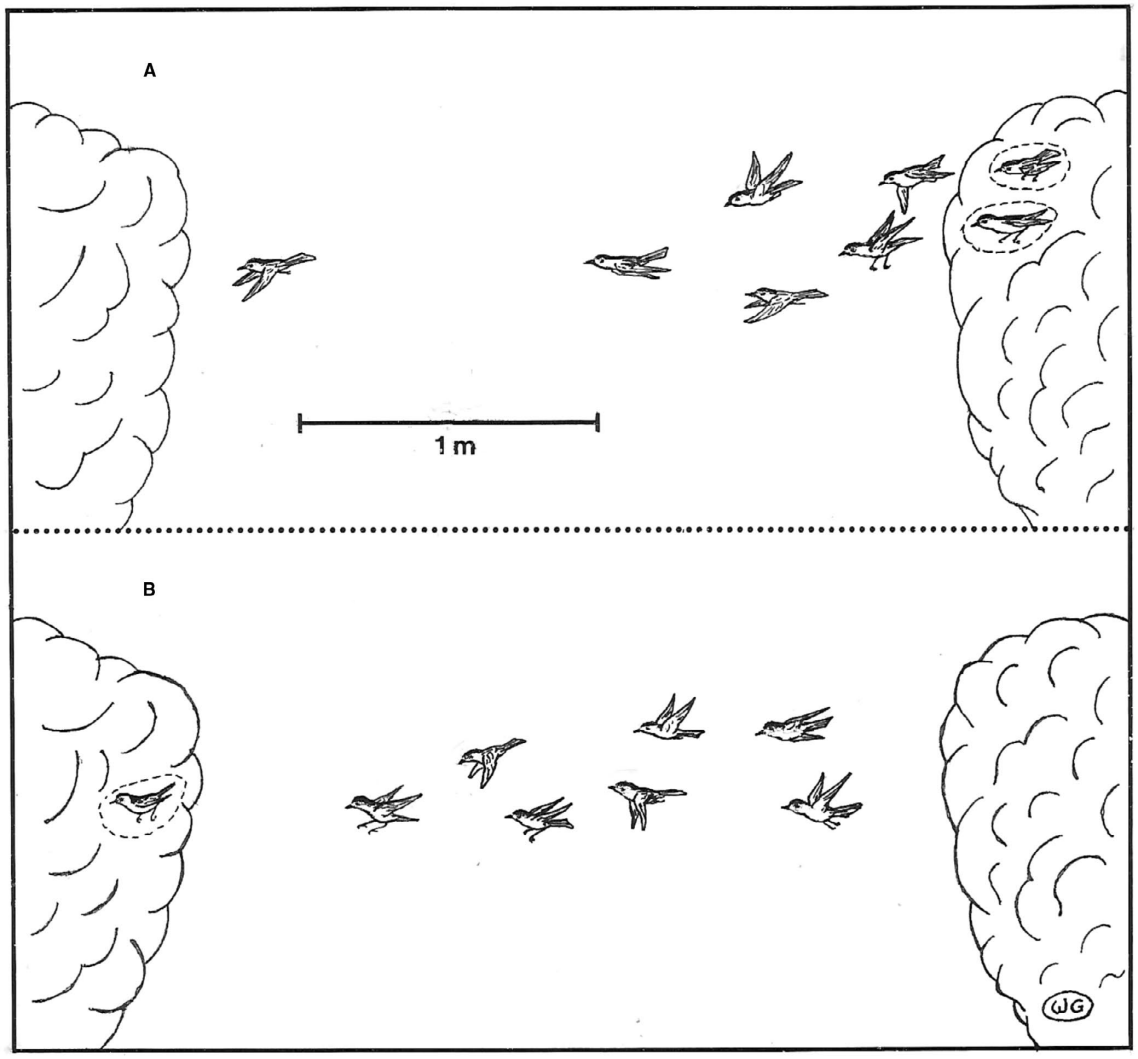
(A) Numbers and dates of migratory return for territorial males, and estimated numbers and dates of migratory return for non‐territorial males. Migratory return numbers and dates for territorial males could be directly determined based on sightings or audio input from male territories. Migratory numbers and dates for non‐territorial males were estimated based on sightings of non‐territorial males over a time length approximately the same as that over which territorial males returned (see text). (B) The corresponding information determined for territorial and non‐territorial females as was determined for territorial and non‐territorial males in (A). (C) Numbers and dates of migratory return for unbanded non‐territorial males sighted over the same time interval as applied in (A). Unbanded non‐territorial male migratory returns are compared with returns for color‐banded non‐territorial males in the same graph, and the comparison serves to test the validity of non‐territorial male identity based on behavior. (D) The corresponding information determined for unbanded and color‐banded females as was determined for unbanded and color‐banded males in (C). The comparison serves to test the validity of non‐territorial female identity based on behavior.

Figure 4A shows both the cumulative number of daily male migratory returns for territorial males, and the cumulative number of daily sightings for an early‐returning sample of non‐territorial males. Figure 4A shows that most territorial males returned from wintering grounds about a month earlier than the early‐detection dates for non‐territorial males. Those early‐detection dates for non‐territorial males are assumed to approximate their migratory return dates. Figure 4B shows the same information for territorial and non‐territorial females and, as with males, most non‐territorial females were first detected, and likely returned from migration, about a month later than did territorial females. Importantly, both Figures 4A,B show evident time gaps between migratory return for most birds in the territorial and non‐territorial subpopulations. These gaps indicate bimodal migratory return for territorials and non‐territorials, that is, territorials and non‐territorials returned in two migratory waves. The visual information presented in Figures 4A,B can be compared with the tabular information presented in Tables 1a,b for males and females, respectively.

### 3.3 Behavior and Proportions of Territorials and Non‐Territorials

#### 3.3.1 Behaviors of Male GANGs in Areas of Female Nest Activity, and in Transient Group Interactions

Males intruding into sites of female nest activity often appeared singly, and I identified many to be color‐banded resident males from nearby territories (Gilbert 2024), to be color‐banded non‐territorial males, or to be unbanded males of undetermined status. At some sites of female nest activity, however, males intruded as closely spaced GANGs. As previously stated in METHODS, evidence indicates that GANGs were composed almost entirely of non‐territorial males, with only one color‐banded territorial male ever observed participating, even though a majority of territorial males were color‐banded. The appearance of GANGs thus served as a prime indicator of migratory return times for non‐territorial males. The behavior of male GANGs at sites of female nest activity was distinctive. The males dispersed closely in undergrowth around the female's location, and if the female flew to a new location several meters away, the males immediately coalesced into a compact (< 0.3 m separation) group, followed the female to her new location, and then dispersed in undergrowth around her. An additional unique GANG behavior observed was “dive bombing.” Some males, upon apparently temporarily losing contact with a targeted female and the other male group members, fluttered above the interacting group, and then fell into the brush that was concealing the group activity.

The behavior of male GANGS in transient group interactions (Ammon and Gilbert 2020) also was distinctive. These groups were not restricted to localized areas of vegetation, as were GANGS intruding into sites of female nest activity. Males in a transient group dispersed closely around a female if she stopped in undergrowth, followed her closely if she progressed slowly through undergrowth (Figure 2), and coalesced into a closely spaced, rapidly pursuing group, resembling a comet, if the female left the undergrowth and flew a great distance away. Females sighted in these long‐distance transient group interactions were not involved in nest building at the times of observation, but nonetheless may have been fertile. As stated, observations of transient group interactions were too infrequent to indicate migratory return dates.

#### 3.3.2 Behavior of Some Females Outside of Breeding Territories

As stated in Section 2, I often sighted individual females in areas of undergrowth outside of, or peripheral to, established Type A breeding territories. I used their distinctive nest activity behaviors, as well as their occurrence in non‐territorial areas, to separate them from territorial females. For extended time periods, non‐territorial females would traverse undergrowth in exploring behavior, and I often saw such females pick up, carry, and then drop vegetation. I never observed these behaviors in territorial females.

#### 3.3.3 Proportions of Banded and Unbanded Territorial Males, and Number and Composition of Sighted GANGS

In this study, 71% (120/169) of all territorial males, including males appearing in their second or later years, were color‐banded. Twenty‐four non‐territorial males also were color‐banded, although that number likely was a small percentage of the total non‐territorial male population. Over the course of the study, I sighted 18 well‐documented occurrences of closely spaced male GANGS intruding into sites of female nest activity, and sighted five GANGS in transient group interactions. The number of males participating in closely spaced male GANGS ranged from three to seven. Among the 18 GANGs sighted intruding into sites of female nest activity, I saw 39 males that definitely lacked color bands, and five males that were color‐banded. Four of the color‐banded males sighted were non‐territorials, and one was territorial. I saw no color‐banded males among the GANGS sighted in the five transient group interactions.

### 3.4 Age Determinations for Territorial and Non‐Territorial Males and Females

Tables 3a,b are contingency tables, for males and females, respectively. Table 3a shows that just two SY males were territory holders. While nearly 90% (17/19) sampled were ASY males. Additionally, 37.5%, (3/8) of ASY male Wilson's Warbler remained non‐territorial into at least their second breeding season. These results indicate that many males did not become territorial, even when they became older. The proportions of SY and ASY territorial and non‐territorial females were similar to those of males, but sample size for females was small. Importantly, however, two females were observed to remain non‐territorial into their second breeding seasons. While the difference in proportional relationship of SY and ASY ages between territorial and non‐territorial males was significant, the difference in proportional relationships for females was not significant.

**TABLE 3:** (a, b) Contingency relationships between territorial status and age of male and female Wilson's Warblers. Proportional relationships for males are statistically significant (*p* = 0.013), but not for females (*p* = 0.190), based on Chi‐squared tests.

	(a) Males		(b) Females	
	Territorial	Non‐territorial	Territorial	Non‐territorial
SY	2	5	4	3
ASY	17	3	7	1

### 3.5 Plotting Migratory Return of Unbanded Versus Color‐Banded Non‐Territorial Samples

Figures 4C,D show plots for early‐encounter dates, assumed to approximate migratory return dates, for unbanded versus color‐banded male and female Wilson's Warblers, respectively. These graphs compare the relative certainty of return dates, based on color‐banded birds, with the return dates for unbanded birds, for which there might be less certainty since those return dates were based on observed behaviors. The graphs, for both males and females, show similar relatively late migratory return periods for both the unbanded and the color‐banded non‐territorial samples.

### 3.6 Natural Mate‐Removal Experiments

I conducted no mate‐removal experiments in this study. However, two “natural” mate‐removal experiments occurred in the study, and in each case, the RF of a pair was left alone to continue breeding after the RM disappeared. In the first case, the RF built a replacement nest in an area seemingly peripheral to, and possibly outside of, the RM and RF's original territory. The original territory remained vacant, the RM was not seen or heard again, and the RF remained unmated. Remaining unmated apparently was not a consequence of the RF being unreceptive to a new mate, however. I played recorded male song in the area of the nest‐building RF on four occasions, and each time the RF approached the recorder and solicited. I otherwise never saw such behavior in a mated female. No male occupied nor sang in the area surrounding the Rf's nest, nor assisted in brood care at her nest. In spite of being unmated, the female uniparentally tended and fledged a brood of four young.

The second natural mate‐removal experiment provided similar results. For 2 days, I observed a color‐banded territorial male guard its mate as she built a replacement nest. However, I did not observe the RM on the third and fourth days of nest building. The RF proceeded to lay and brood four eggs, plus brood one egg of a Brown‐headed Cowbird (*Molothrus ater*). The female uniparentally fed the four Wilson's Warbler nestlings and the one cowbird chick. The cowbird fledged, and the four warbler chicks were ejected. No new male occupied the territory in which the nest was located, nor assisted in brood care at the nest. About a month after nest building had been initiated, however, I observed a Wilson's Warbler in the territory with a missing leg, and the other leg banded with a F & W band. I assume that this injured bird was the RM, and that it had abandoned its mate, nest, and brood, because of its leg injury. Since I had banded the male in the previous year, it apparently required over a year for the leg injury to develop, presumably from irritation from the color bands.

Although the sample size of these natural mate‐removal experiments is small, they suggest that Wilson's Warbler males returning later from migration do not take over Type A breeding territories that may become vacant, and do not pair with territorial females that may become available. Stated another way, all later‐returning male Wilson's Warblers become and remain non‐territorial.

## 4 Discussion

### 4.1 Non‐territorial Wilson's Warblers: Migratory Return Dates and Ages

#### 4.1.1 Migratory Return Dates

This study determined that non‐territorial male and female Wilson's Warblers returned from wintering grounds about 3 to over 5 weeks later than did respective sexes of territorials, depending on variable methods of determining mean return dates (Tables 1a–d). These later‐returning non‐territorials constituted a second migratory wave composed entirely of non‐territorials. These findings are the same as the bimodal migratory return for Wilson's Warblers reported by Stewart 1973, and the second waves in both studies might be adhering to cost‐minimizing dates hypothesized by Kokko (1999) for migratory birds.

#### 4.1.2 Ages of Non‐Territorials

I recorded just two male Wilson's Warblers becoming territorial in their first (SY) breeding season (Table 3a). I recorded no SY female initially becoming territorial in its first (SY) breeding season, although few were aged. Several SY females did become mates of territorial males that apparently lost their original mates. Thus, nearly all SY males, and most SY females in my study population were non‐territorials. I am not aware that such reduced levels of territoriality, among SY males and females, have been reported for other studied passerine species (e.g., Bayne and Hobson 2002; Betts et al. 2008; Cooper et al. 2009), albeit territories of SY birds in those other passerine species often were reported to be in less‐suitable breeding habitat than were the territories of ASY birds.

### 4.2 Different Behaviors and Group Compositions in Territorial and Non‐Territorial Subpopulations of Male Wilson's Warblers

#### 4.2.1 Male Behaviors

I have reported that later‐returning, non‐territorial Wilson's Warbler males in my study population displayed different behaviors than did earlier‐returning territorials. To summarize, later‐returning males never attempted to form Type A breeding territories characteristic of earlier‐returning males. Also, later‐returning males seldom sang, at least early in the breeding season, whereas territorial males sang persistently if unmated, or when defending territorial borders (Ammon and Gilbert 2020). Also, non‐territorial males frequently formed GANGS of closely spaced (< 0.3 m) individuals when pursuing females, whereas territorial males never formed such GANGS.

#### 4.2.2 Composition of Male GANGs, Based on Proportions of Banded and Unbanded Birds

The closely spaced male GANGs sighted intruding into areas of female nest activity, as well as in farther‐ranging male transient group interactions, were composed entirely, or mostly, of unbanded birds. Also, with one exception, the few color‐banded males sighted in GANGs were non‐territorials. This paucity of color‐banded birds seen in GANGs indicates that such GANGs likely were not primarily composed of territorial males, since 71% (120/169) of territorial males tabulated over years were color banded. Additionally, no such closely spaced male GANG likely was composed of unbanded territorial males flying in from outside of the study area, since a prior study (Gilbert 2024) found that no color‐banded territorial male intruded into breeding territories from distances greater than 200 m, and about 88% of intrusions were from nearby territories at distances of 100 m or less. Finally, while only about 20% (24/117) of all males color‐banded in the study area were non‐territorials, those banded non‐territorial males comprised 80% (4/5) of all color‐banded males seen to be members of GANGs intruding into areas of nest activity. In sum, evidence indicates that the 18 closely spaced intruding male GANGs that I sighted, and the five transient group interactions that I sighted, likely were composed entirely, or almost entirely, of non‐territorial males.

### 4.3 Dual Waves of Migratory Return of Wilson's Warblers, and Migratory Return of ASY and SY Birds in Other Species

#### 4.3.1 Time Lengths Between Migratory Waves of Wilson's Warblers

Stewart (1973) reported that Wilson's Warblers returned to his two study sites in two migratory waves. In his study site #1, the earlier wave, composed of five ASY males, returned 9–12 days earlier than did a single later returning male of unknown age. In his study site #2, six unaged males returned 17–21 days earlier than did three later males. For the combined study sites, the mean return date for the initial migratory waves was 23 March ±4.5 days SD, and the mean return date for the later migratory waves was 13 days later on 5 April ±0.8 days SD. The difference in mean migratory return dates was statistically significant (*p* < 0.0001).

Visual inspection from the current study shows that male and female Wilson's Warblers in my study population also returned to breeding grounds in two migratory waves (Figures 4A,B), with a distinct gap between the two waves. The time intervals determined between the means of these migratory waves of territorial and non‐territorial samples, were 31 days for males and 28 days for females (Tables 1a,b). The mean difference was 22.4 days based on samples of just the five earliest‐arriving territorial and non‐territorial males (Table 1c), and 38.6 days, based on samples of seven males when they were territorial, versus the same males when they were non‐territorial (Table 1d). Differences between all respective earlier and later mean migratory return dates were statistically significant.

The time lengths between return of migratory waves of territorial and non‐territorial Wilson's Warbler determined in this study, varying between about 3 and 5 weeks, were longer than the approximate 2‐week difference determined by Stewart (1973). However, Stewart's study involved much‐reduced sample sizes, compared with this study. Even so, Stewart's (1973) results still show differences in mean migratory return dates that, for the most part, are greater than differences determined for subpopulations of other passerine species (see below).

#### 4.3.2 Time Lengths Between Migratory Return for ASY and SY Subpopulations of Other Passerine Species

No field study, to the best of my knowledge, has separated migratory arrival dates for territorial and floater subpopulations of a migratory passerine species. The closest approximations to migratory return dates for territorial and floater subpopulations of such passerines are seen in migratory return dates for ASY and SY subpopulations. In that regard, differences between mean migratory return times for earlier and later waves of migratory return for Wilson's Warblers, determined in this study and that of Stewart (1973), were considerably greater than the mean time interval of just 3–4 days between ASY and SY birds reported by Stewart, Francis, and Massey (2002), for samples of 20 different passerine species passing through an eastern US banding station. It seems possible, however, that the passage times for birds through banding stations during migration might not be completely indicative of their later arrival times on breeding grounds. For example, if SY birds required greater times to migrate toward breeding grounds than ASY birds, then passage time differences of a few days at banding stations could extend to greater time intervals before reaching breeding grounds. However, the intervals for arrival times of Wilson's Warbler migratory waves determined in this study, and that of Stewart (1973), also are greater than mean arrival time differences between age classes that have been determined at breeding grounds. These breeding grounds arrival differences are about 7 days between ASY and SY subpopulations for Eastern Kingbirds (*Tyrannus tyrannus*) reported by Cooper et al. (2009), are a 6–10 days mean difference reported by Hahn and Silverman (2006), and are a 8–14 days mean difference reported by Lozano, Perreault, and Lemon (1996), for ASY and SY subpopulations of American Redstarts ( *Setophaga ruticilla* ). The findings for Wilson's Warblers also are mostly greater than the “approximately two weeks” ASY/SY mean migratory return time difference for Black‐headed Grosbeaks ( *Pheucticus melanocephalus* ) determined by Hill (1988). Additional to the generally shorter mean migratory return times between ASY and SY subpopulations reported for multiple passerine species, compared with mean return times for subpopulations of Wilson's Warblers, there are no reports that the other passerine species returned in two migratory waves, as is the case for Wilson's Warblers. This absence of reports of distinct migratory waves for other studied passerine species may be relevant. Subpopulations of older and younger birds, returning to breeding grounds at different mean dates, still could return in what might be considered a single migratory wave, with wide temporal variance in the two overlapping subpopulations, and not in two migratory waves, with distinct temporal separation between the two waves, as is the case for Wilson's Warblers.

#### 4.3.3 Flexibility in Self‐Perceived Adaptive Value of Territoriality Versus Non‐Territoriality, and in Early Versus Later Migratory Return, Among Males

Even given an evolved greater adaptive value for early migratory return and territoriality over later migratory return and non‐territoriality in most passerine species, evidence from this study indicates that adaptive values for these two behaviors varied among different male Wilson's Warblers (Table 1c), and within the same males among different years (Table 1d). These results indicate that some different males, and the same males at different times, “chose” later migratory return, and thus non‐territoriality, as preferred options to earlier migratory return, and possible territoriality. Such variation in choice likely depended on such factors as age, physical and physiological condition, and energy reserves of individual males. Observations from this study also indicate that “experience” may contribute to self‐perceived adaptive value of early migratory and territoriality versus later migratory return and non‐territoriality. Two color‐banded males that originally were territorial, but were displaced from their territories by other males, subsequently became non‐territorial, and were encountered relatively late as non‐territorials during each of their remaining years in the study area (status category 5, stated above). I hypothesize that the initial failures at territoriality of these two males may have contributed to “decisions” to subsequently become non‐territorial. Since decisions to return early or late to breeding grounds, and to become territorial or non‐territorial, cannot be made based on prior knowledge of conditions on breeding grounds or during migratory passage, it follows that such decisions must be based on endogenous conditions, be they anatomical, physiological, or “psychological,” as they pre‐exist for individual birds on wintering grounds.

#### 4.3.4 Adaptive Value of Non‐Territorial Behavior in Another Passerine

The only previous study showing greater adaptive value for non‐territoriality over territoriality, of which I am aware, is that of Theimer et al. (2018) for Southwestern Willow Flycatchers (Empidonax traillii *extimus* ). Individual birds that remained as floaters, during a year of exceptional drought, had greater survival and annual productivity in subsequent years than did individuals that attempted territoriality during the drought year. Although the environmental circumstances leading to more adaptive non‐territoriality for Willow Flycatchers during 1 year were exceptional, the results of the study do demonstrate that non‐territoriality in a territorial species sometimes can be more adaptive than territoriality. I had no evidence, however, that unusual environmental conditions contributed to later migratory return and non‐territoriality in my non‐territorial subpopulations of Wilson's Warblers.

### 4.4 Adaptive Value of Late Migratory Return, and Non‐Territoriality, in Long‐Lived Bird Species, and Possible Exception for a Short‐Lived Species

For long‐lived bird species, extended time intervals between waves of migratory return frequently have been found, and make adaptive sense. Later returnees often are younger birds, and there apparently is adaptive value from gaining breeding‐grounds' experience, rather than initially trying to compete with older, more‐experienced birds for territories (Penteriani, Ferrer, and Delgado 2011; Tanferna et al. 2013). For short‐lived migratory, territorial species, however, and with the possible exception of unusual environmental circumstances such as reported by Theimer et al. (2018), returning to breeding grounds substantially later than an initial migratory wave, and not trying to compete for a territory, seems not to make adaptive sense. Such short‐lived species have a limited number of breeding seasons, often just one, to pass on their genes, and competing for breeding territories, even as SY birds, would seem evolutionarily essential (Lenda, Maciusik, and Skórka 2012). Thus, no short‐lived territorial bird species should have evolved behaviors, such as late migratory return, which essentially forfeits their initial breeding seasons. Wilson's Warbler migratory return in two waves, as determined by Stewart (1973) and this study, thus superficially makes no adaptive sense. However, the paradox, and possible implications, of Stewart's (1973) findings of dual migratory waves, were overlooked by subsequent empirical and theoretical work, until the current study. Stewart's (1973) findings indirectly indicate that territoriality may not be absolutely essential for substantial reproductive success in Wilson's Warblers, and findings from this study indicate the same thing. Further information regarding possible adaptive values associated with Wilson's Warbler later migratory return, and non‐territoriality, should be of interest.

## Author Contributions

**William M. Gilbert:** conceptualization (equal), data curation (equal), formal analysis (equal), funding acquisition (equal), investigation (equal), project administration (equal), visualization (equal), writing – original draft (equal), writing – review and editing (equal).

## Conflicts of Interest

The author declares no conflicts of interest.

## Data Availability

Data archived with Dryad under https://doi.org/10.5061/dryad.k98sf7mgz.
